# Analysis of Subthreshold Current Reset Noise in Image Sensors

**DOI:** 10.3390/s16050663

**Published:** 2016-05-10

**Authors:** Nobukazu Teranishi

**Affiliations:** 1 Research Institute of Electronics, Shizuoka University; 3-5-1 Johoku, Naka-ku, Hamamatsu 432-8011, Japan; teranishi@idl.rie.shizuoka.ac.jp; Tel.: +81-53-478-1313; 2 Laboratory of Advanced Science and Technology for Industry, University of Hyogo; 1-1-2 Koto, Kamigori, Ako-gun, Hyogo 678-1205, Japan

**Keywords:** CMOS image sensor, 3-transistor scheme, reset noise, subthreshold current, hard reset, soft reset, feedback reset, tapered reset

## Abstract

To discuss the reset noise generated by slow subthreshold currents in image sensors, intuitive and simple analytical forms are derived, in spite of the subthreshold current nonlinearity. These solutions characterize the time evolution of the reset noise during the reset operation. With soft reset, the reset noise tends to $\sqrt{mkT/2C_{PD}}$ when t→∞, in full agreement with previously published results. In this equation, $C_{PD}$ is the photodiode (PD) capacitance and *m* is a constant. The noise has an asymptotic time dependence of $t^{-1}$, even though the asymptotic time dependence of the average (deterministic) PD voltage is as slow as logt. The flush reset method is effective because the hard reset part eliminates image lag, and the soft reset part reduces the noise to soft reset level. The feedback reset with reverse taper control method shows both a fast convergence and a good reset noise reduction. When the feedback amplifier gain, *A*, is larger, even small value of capacitance, $C_{P}$, between the input and output of the feedback amplifier will drastically decrease the reset noise. If the feedback is sufficiently fast, the reset noise limit when t→∞, becomes $\frac{mkT{\left(C_{PD}+C_{P1}\right)}^{2}}{2q^{2}A\left(C_{PD}+\left(1+A\right)C_{P}\right)}$ in terms of the number of electron in the PD. According to this simple model, if *C_PD_* = 10 fF, *C_P_*/*C_PD_* = 0.01, and *A* = 2700 are assumed, deep sub-electron rms reset noise is possible.

## 1. Introduction

Four-transistor (4-Tr) complementary metal-oxide-semiconductor (CMOS) image sensors [1] are widely used in various applications, such as mobile phone cameras, digital still cameras, security, industrial, medical equipment, *etc.* They have significant advantages compared with three-transistor (3-Tr) CMOS image sensors. Firstly, the 4-Tr scheme can use pinned photodiodes (PPDs) [2,3,4,5,6] to reduce the dark current. Secondly, the complete charge transfer by the PPD [2] realizes “first reset, later signal” and correlated double sampling (CDS) [7], which eliminates both the reset noise at the floating diffusion node and the low frequency noise at the source follower amplifier. Thirdly, the capacitance of the floating diffusion can be decreased by fine processing technology and a large conversion gain can be obtained, which increases the signal-to-noise ratio. Fourthly, the shared transistor technology [8,9] reduces the number of transistors per pixel. The minimum reported transistor number per pixel is 1.375 transistors/pixel [10], which is much smaller than that of 3-Tr scheme.

The 3-Tr scheme is now being used for large pixel CMOS image sensors. One example is its use in medical X-ray image sensors. The typical pixel size is around 100 μm. There are several reasons why the 3-Tr scheme is being used. The first one is that it is difficult to achieve a complete charge transfer of the PPD with such large pixels or PDs. Another one lies in the fact that X-ray image sensors usually suffer from photon shot noise and the readout noise of the 3-Tr scheme is acceptable. A third reason is that the fabrication process for the 3-Tr scheme is simpler than that of the 4-Tr scheme. A fourth reason is the fact that the 3-Tr scheme can be operated in non-destructive readout mode, and can realize dose sensing during radiation or auto exposure control (AEC) using fast-frame-rate skip mode [11]. Finally, a fifth reason is that the 3-Tr scheme can reach a higher number of saturation electrons at the PD than that of the 4-Tr scheme.

If the 3-Tr scheme were able to achieve low readout noise, it could be used in more applications, in particular, elevated image sensors, or photosensitive material hybrid image sensors, which cannot use the PPD complete charge transfer scheme. Some organic photoconductive films have larger absorption coefficients than that of silicon, and smaller photosensitive layer thicknesses can provide enough sensitivity. Crosstalk could then be reduced even for small pixel size, and elevated image sensors with organic photoconductive films would become candidates for small pixel image sensors [12,13]. Elevated image sensors can have sensitivities beyond the silicon sensitive wavelength range, well within the ultraviolet (UV) and infrared (IR) range. For example, crystal selenium (c-Se) has a 1.74 eV bandgap and is a good sensitive material for both UV and visible light [14]. Germanium (Ge) and indium-gallium-arsenide (InGaAs) have 0.8 eV and 0.36–1.43 eV direct bandgaps, respectively, and are good photosensitive materials for near IR [15,16]. These hybrid image sensor developments might be accelerated by recent advances in 3D and hybrid technology.

**Figure 1:**
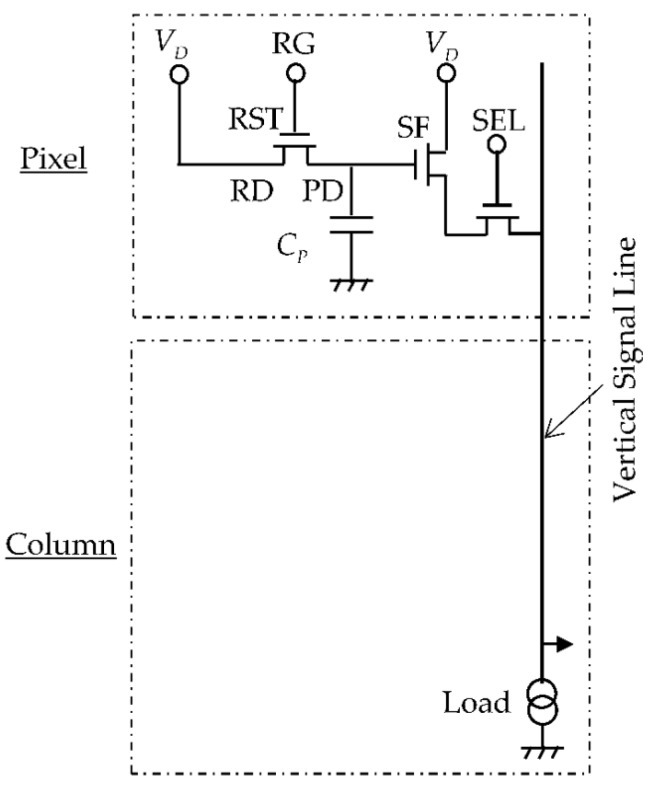
Pixel and column schematic for 3-Tr scheme CMOS image sensor.

A 3-Tr pixel consists of an N-type PD, a reset transistor (RST) to reset PD, a source follower amplifier (SF) which picks up the PD voltage and sends the voltage signal to the column circuit, and a select transistor (SEL) which activates the selected row, as shown in Figure 1. The reset noise of the PD is the dominant noise source in the 3-Tr scheme. The original reset method is hard reset. Its noise variance is calculated as *kTC* [17,18], where *k* is the Boltzmann constant, *T* the absolute temperature, and *C* the detection capacitance. This noise is therefore called “*kTC* noise”. Various other reset methods have been proposed to reduce the reset noise and will be discussed later soft reset [19,20], feedback reset [21,22], feedback reset with taper control [13,23,24,25,26]. Feedback reset has realized a reset noise level as small as 2.9 e^−^·rms (electrons rms) [13]. While those approaches aim to reduce the reset noise itself, other approaches to the problem have been attempted; one of them is to reduce effective detection capacitance, thus increasing signal voltage. For this purpose, a charge sensitive amplifier or capacitive transimpedance amplifier is introduced [27,28]. Another approach is to introduce in-pixel CDS [29].

In this paper, we will discuss the reset noise reduction itself. A fundamental time-domain analysis of various reset methods is presented, and the reset noise is studied in detail. In the next section, our reset noise analysis technique is introduced. In Section 3, Section 4, Section 5 and Section 6, the hard reset, soft reset, tapered reset, and feedback reset with reverse taper control methods will be analyzed. In Section 7, the possibility of photon counting by the 3-Tr scheme is discussed.

## 2. Reset Noise Analysis Technique

To discuss the various reset methods, a reset noise analysis technique must be prepared, preferably one capable of providing an intuitive and simple analytical solution without numerical or Monte Carlo simulations in spite of the nonlinearity of the subthreshold current. The subthreshold current causes a slow reset operation, therefore, the time dependence of the reset noise during the reset operation period needs to be evaluated, from the initial condition to the final state.

A frequency domain analysis has previously been published, where the estimated reset noise was compared with measurement result [26]. The steady-state noise (final stage noise) was calculated using a resistor instead of the reset transistor. A time domain analysis was proposed, using effectively-second-order differential equation [25]. To derive a closed form expression, a fixed resistance was also used instead for the reset transistor. Another time domain method was proposed for soft reset analysis, directly treating the subthreshold current nonlinearity and assuming the existence of a shot noise in the subthreshold current [20]; it obtained a soft reset noise of *kT/2C*, which agrees well with the measurements. However, it would be desirable that improved reset methods such as feedback reset with taper control could also be analytically treated.

In the rest of this section, our reset noise analysis is introduced. The PD node voltage *V_PD_*(*t*) is decomposed into a deterministic (or average) part *V_PDa_*(*t*) and a stochastic (or noise) part *v_PD_*(*t*). Naturally:

(1) $$V_{PD}\left(t\right)=V_{PDa}\left(t\right)+v_{PD}\left(t\right)$$

To derive the analytical form of the reset noise variance $<v_{PD}{\left(t\right)}^{2}>$ three steps are needed in this analysis:

Step 1:  The equation for the average part *V_PDa_*(*t*) is derived, and the solution is obtained.
Step 2:  The equation for the noise part *v_PD_*(*t*) is derived, and *v_PD_*(*t*) is obtained explicitly.
Step 3:  The variance $<v_{PD}{\left(t\right)}^{2}>$ is calculated.

This approach is straightforward and logically simple. In the following sections, this analysis is applied to hard reset, soft reset, tapered reset and feedback reset with taper control.

## 3. Hard Reset

**Figure 2:**
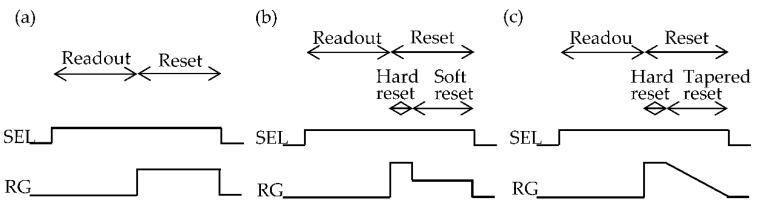
Timing diagram for one pixel. (**a**) Hard reset and soft reset; (**b**) Flush reset; and (**c**) Tapered reset.

Hard reset is originally applied in the reset of the floating diffusion of CCD (Charge coupled device), and is the original reset method of 3-Tr CMOS image sensor. Its timing diagram is shown in Figure 2a. The hard reset noise variance was derived as *kTC* using frequency domain analysis [17] and time domain analysis [18]. The same result will be derived here.

With hard reset, the RST channel can be regarded as a pure resistance *R*, because the RST operates in the linear region. Resistances generate Johnson noise or thermal noise, from which the reset noise arises. To simplify the model, it is also assumed that there is no dark current or no incident light during the reset phase. This assumption is also used in Section 4, Section 5 and Section 6. The continuity equation is:

(2) $$C_{PD}\frac{dV_{PD}\left(t\right)}{dt}=\frac{V_{RD0}-V_{PD}\left(t\right)}{R}+i_{n}\;\left(t\right)$$

where *C_PD_* is the PD capacitance, *V_RD0_* is the reset transistor drain (RD) voltage, and *i_n_*(*t*) is the thermal noise associated with resistance *R*, whose autocorrelation is:

(3) $$<i_{n}\left(t_{1}\right)i_{n}\left(t_{2}\right)>=\frac{2kT}{R}\delta \left(t_{1}-t_{2}\right)$$

When applied at Step 1, the equation of continuity becomes:

(4) $$C_{PD}\frac{dV_{PDa}\left(t\right)}{dt}=\frac{V_{RD0}-V_{PDa}\left(t\right)}{R}$$

The solution is obtained as:

(5) $$V_{PDa}\left(t\right)=V_{PDa}\left(0\right)e^{-\frac{t}{\tau_{HR}}}+V_{RD0}\left(1-e^{-\frac{t}{\tau_{HR}}}\right)$$

where time constant, $\tau_{HR}$ is given by:

(6) $$\tau_{HR}\equiv C_{PD}R$$

If the parameters of a typical RST are assumed, with a 0.4 μm channel width, 0.55 μm channel length, 6 nm thick gate oxide, $V_{GS}=3.3\text{ V}$, and $V_{RD0}=3.3\;V$, we will have R≈10 kΩ. For $C_{PD}$ = 10 fF, $\tau_{HR}$ becomes 100 ps, which is much smaller than the typical reset period, 1 μs. When t→∞, $V_{PDa}\left(t\right)$ converges to $V_{RD0}$.

For Step 2, we substitute Equation (5) into Equation (2), to obtain the equation for $v_{PD}\left(t\right)$ as:

(7) $$C_{PD}\frac{dv_{PD}\left(t\right)}{dt}=\frac{v_{PD}\left(t\right)}{R}+i_{n}\;\left(t\right)$$

The solution of this equation is:

(8) $$v_{PD}\left(t\right)=\frac{1}{C_{PD}}\int_{o}^{t}dt^{\prime }e^{\frac{t^{\prime }-t}{\tau_{HR}}}i_{n}\left(t\prime \right)+e^{-\frac{t}{\tau_{HR}}}v_{PD}\left(0\right)$$

Finally, for Step 3, we square Equation (8) to obtain:

(9) $$v_{PD}{\left(t\right)}^{2}=\frac{1}{C_{PD}{}^{2}}\int_{o}^{t}\int_{0}^{t}dt_{1}dt_{2}e^{\frac{t_{1}-t}{\tau_{HR}}+\frac{t_{2}-t}{\tau_{HR}}}i_{n}\left(t_{1}\right)i_{n}\left(t_{2}\right)+\frac{2e^{-\frac{t}{\tau_{HR}}}v_{PD}\left(0\right)}{C_{PD}}\int_{o}^{t}dt_{1}e^{\frac{t_{1}-t}{\tau_{HR}}}i_{n}\left(t_{1}\right)+e^{-\frac{2t}{\tau_{HR}}}v_{PD}{}^{2}\left(0\right)$$

Averaging Equation (9) and using Equation (3), the hard reset noise variance is obtained as:

(10) $$<v_{PD}{\left(t\right)}^{2}>=\frac{kT}{C_{PD}}\left(1-e^{-\frac{2t}{\tau_{HR}}}\right)+<v_{PD}{\left(0\right)}^{2}>e^{-\frac{2t}{\tau_{HR}}}$$

The first term is caused by thermal noise, and the second term comes from the initial condition. Because of the exponential decay, the reset noise variance $<v_{PD}{\left(t\right)}^{2}>$ is sufficiently settled within the reset period. When *t* →∞:

(11) $$<v_{PD}{\left(\infty \right)}^{2}>=\frac{kT}{C_{PD}}.$$

The well-known *kTC* noise is therefore produced.

## 4. Soft Reset

The soft reset method was introduced to reduce the reset noise. Even though the timing diagram is the same as that of the hard reset, the RG (RST gate) on voltage is smaller. With the soft reset method, the RST is operated first in the saturation region, and then in the subthreshold region. The signal charge transfer from the PD to the RD in the saturation region is smooth and the period is as small as a few nanoseconds, it does not substantially contribute to the reset noise, when compared with the following subthreshold region period. The reset noise will therefore be calculated neglecting the saturation period, using only subthreshold region period; *t* = 0 in this analysis corresponds to the moment when RST enters this region.

The equation of continuity then becomes:

(12) $$C_{PD}\frac{{dV}_{PD}\left(t\right)}{dt}=I_{a}\left(t\right)+i_{n}\;\left(t\right)=I_{0}e^{-{\beta V}_{PD}\left(t\right)}+i_{n}\;\left(t\right)$$

where $I_{a}\left(t\right)$ is the average drain current, $I_{0}$ is a constant, β≡q/mkT, $m\equiv 1+C_{D}/C_{G}\;$, $C_{D}$ is the depletion-layer capacitance and $C_{G}$ is the gate capacitance. Typically, *m* is slightly above 1. The subthreshold current has shot noise with autocorrelation:

(13) $$<i_{n}\left(t_{1}\right)i_{n}\left(t_{2}\right)>={qI}_{a}\left(t\right)\delta \left(t_{1}-t_{2}\right)$$

For Step 1, the continuity equation for the average voltage $V_{PDa}\left(t\right)$ is given by:

(14) $$C_{PD}\frac{dV_{PDa}\left(t\right)}{dt}=I_{0}e^{-\beta V_{PDa}\left(t\right)}$$

Even though this equation is nonlinear, it has an analytical solution, which can be obtained with the variation of parameters method. The solution [2] is:

(15) $$V_{PDa}\left(t\right)=\frac{1}{\beta }log\left[e^{\beta V_{PDa}\left(0\right)}+\frac{t}{\tau }\right]$$

where:

(16) $$\tau \equiv C_{PD}/\beta I_{0}$$

The existence of this analytical solution is essential for the subthreshold current reset noise analyses. When t→∞, $V_{PDa}\left(t\right)$ diverges slowly as a logarithmic function. The soft reset has no finite limit, even though the hard reset has $V_{RD0}$ as a limit. This is an important characteristic for the soft reset.

For Step 2, we substitute Equation (15) into Equation (12) and obtain the equation for $v_{PD}\left(t\right)$:

(17) $$C_{PD}\frac{dv_{PD}\left(t\right)}{dt}=-I_{0}e^{-\beta V_{PDa}\left(0\right)}\frac{1-e^{-\beta v_{PD}\left(t\right)}}{1+\frac{t}{\tau }e^{-\beta V_{PDa}\left(0\right)}}+i_{n}\;\left(t\right)$$

Considering that $\beta v_{PD}\left(t\right)\;\ll 1,$ the approximation, $e^{-\beta v_{PD}\left(t\right)}\approx 1-\beta v_{PD}\left(t\right)$ can be used. Equation (17) then becomes a linear equation:

(18) $$C_{PD}\frac{dv_{PD}\left(t\right)}{dt}=-\frac{\beta I_{0}e^{-\beta V_{PDa}\left(0\right)}}{1+\frac{t}{\tau }e^{-\beta V_{PDa}\left(0\right)}}v_{PD}\left(t\right)+i_{n}\;\left(t\right)$$

Its solution is given by:

(19) $$v_{PD}\left(t\right)=\frac{1}{C_{PD}}\int_{o}^{t}dt\prime \frac{1+\frac{t\prime }{\tau }e^{-\beta V_{PDa}\left(0\right)}}{1+\frac{t}{\tau }e^{-\beta V_{PDa}\left(0\right)}}i_{n}\left(t\prime \right)+\frac{1}{1+\frac{t}{\tau }e^{-\beta V_{PDa}\left(0\right)}}v_{PD}\left(0\right)$$

For Step 3, squaring Equation (19), averaging and using Equation (13), the soft reset noise variance can be obtained:

(20) $$<v_{PD}{\left(t\right)}^{2}>=\frac{mkT}{2C_{PD}}\left(1-\frac{1}{{\left(1+\frac{t}{\tau }e^{-\beta V_{PDa}\left(0\right)}\right)}^{2}}\right)+<v_{PD}{\left(0\right)}^{2}>\frac{1}{{\left(1+\frac{t}{\tau }e^{-\beta V_{PDa}\left(0\right)}\right)}^{2}}$$

Using the fact that $I\left(0\right)=I_{0}e^{-\beta V_{PDa}\left(0\right)}$, Equation (20) can be rewritten as:

(21) $$<v_{PD}{\left(t\right)}^{2}>=\frac{mkT}{2C_{PD}}\left(1-\frac{1}{{\left(1+\frac{qI_{a}\left(0\right)t}{mkTC_{PD}}\right)}^{2}}\right)+<v_{PD}{\left(0\right)}^{2}>\frac{1}{{\left(1+\frac{qI_{a}\left(0\right)t}{mkTC_{PD}}\right)}^{2}}$$

The first term is caused by shot noise, and the second term results from the initial condition. When t→∞, the asymptotic form and the limit are obtained as:

(22) $$<v_{PD}{\left(t\right)}^{2}>\approx \frac{mkT}{2C_{PD}}\left(1-{\left(\frac{mkTC_{PD}}{qI_{a}\left(0\right)t}\right)}^{2}\right)+<v_{PD}{\left(0\right)}^{2}>{\left(\frac{mkTC_{PD}}{qI_{a}\left(0\right)t}\right)}^{2}$$

(23) $$<v_{PD}{\left(t\right)}^{2}>\to \frac{mkT}{2C_{PD}}$$

It should be noted that the asymptotic time dependence of the noise standard deviation $\sqrt{<v_{PD}{\left(t\right)}^{2}>}$ behaves as $t^{-1}$ although the asymptotic time dependence of the average PD voltage, $V_{PDa}\left(t\right)$ behaves as logt (as shown in Equation (15)), which is much slower than $t^{-1}$. In the hard reset case, $V_{PDa}\left(t\right)$ and $\sqrt{<v_{PD}{\left(t\right)}^{2}>}$ have the same exponential time dependence (with $e^{-t/\tau_{HR}}$). The determinant time constant in Equations (21) and (22), $\tau_{SR}\equiv mkTC_{PD}/\;qI\left(0\right)$, is calculated for a typical case, as follows. Assuming that $C_{PD}=10\text{ fF}$, $I_{a}\left(0\right)=0.5\;\mu \text{A}$, $v_{th}\equiv kT/q=26\text{ mV }\left(\text{at }300\text{ K}\right),$ m=1, we have that $\tau_{SR}=0.52\text{ ns}$. It is small enough when compared with the typical reset period, 1 μs. The limit at t→∞ is *mkT*/2*C_PD_*, which fits the results obtained in previous works [19,20,30,31,32,33].

To alleviate the image lag problem of soft reset image sensors [2], the flushed reset method was proposed [20,22,33]. In this method, during one reset period, a hard reset is first carried out to eliminate vestige of the previous signal, and a soft reset is then performed to reduce the reset noise. The timing chart for a simple case of the flushed reset method is shown in Figure 2b. The reset noise variance after the hard reset is $kT/C_{PD}$, as given as Equation (11), and this becomes the initial condition for the soft rest period. Substituting $<v_{PD}{\left(0\right)}^{2}>=kT/C_{PD}$ into Equation (21), the flushed reset noise can be derived as:

(24) $$<v_{PD}{\left(t\right)}^{2}>=\frac{mkT}{2C_{PD}}\left(1+\left(\frac{2}{m}-1\right)\frac{1}{{\left(1+\frac{qI\left(0\right)t}{mkTC_{PD}}\right)}^{2}}\right)$$

If the reset period is enough long, $<v_{PD}{\left(t\right)}^{2}>$ becomes:

(25) $$<v_{PD}{\left(t\right)}^{2}>\to \frac{mkT}{2C_{PD}}$$

The hard reset part eliminates image lag, and the soft reset part reduces the reset noise to the soft reset level.

## 5. Tapered Reset

To improve the convergence at the soft reset method, tapered reset is proposed. In this method, the RST gate voltage is gradually decreased to 0 V during the soft reset period, as shown in Figure 2c. The continuity equation becomes:

(26) $$C_{PD}\frac{dV_{PD}\left(t\right)}{dt}=I_{a}\left(t\right)+i_{n}\;\left(t\right)=I_{0}e^{-\beta V_{PD}\left(t\right)-\beta at}+i_{n}\;\left(t\right)$$

where *a* is a positive constant characterizing the slope of the RST taper, in unit of V/s.

For Step 1, the continuity equation for average voltage $V_{PDa}\left(t\right)$ is:

(27) $$C_{PD}\frac{dV_{PDa}\left(t\right)}{dt}=I_{0}e^{-\beta V_{PDa}\left(t\right)-\beta at}$$

Its solution is:

(28) $$V_{PDa}\left(t\right)=\frac{1}{\beta }log\left[e^{\beta V_{PDa}\left(0\right)}+\frac{I_{0}}{C_{PD}a}\left(1-e^{-\beta at}\right)\right]$$

When t→∞, $V_{PDa}\left(t\right)$ converges to:

(29) $$V_{PDa}\left(\infty \right)=\frac{1}{\beta }log\left[e^{\beta V_{PDa}\left(0\right)}+\frac{I_{0}}{C_{PD}a}\right]$$

As seen, while $V_{PDa}\left(t\right)$ for the soft reset diverges slowly as logarithmic function, that of the tapered reset converges exponentially to a constant; this happens because the drain current is extinguished as a consequent of the taper control.

For Step 2, substituting Equation (28) into Equation (26), the equation for $v_{PD}\left(t\right)$ can be obtained as:

(30) $$C_{PD}\frac{dv_{PD}\left(t\right)}{dt}=-I_{0}e^{-\beta V_{PDa}\left(0\right)-\beta at}\frac{1-e^{-\beta v_{PD}\left(t\right)}}{1+\frac{I_{0}e^{-\beta V_{PDa}\left(0\right)}}{C_{PD}a}\left(1-e^{-\beta at}\right)}+i_{n}\;\left(t\right)$$

Considering that $\beta v_{PD}\left(t\right)\;\ll 1,$ the approximation $e^{-\beta v_{PD}\left(t\right)}\approx 1-\beta v_{PD}\left(t\right)$ can be used. Equation (30) then becomes a linear equation:

(31) $$C_{PD}\frac{dv_{PD}\left(t\right)}{dt}=-\frac{\beta I_{0}e^{-\beta V_{PDa}\left(0\right)-\beta at}}{1+\frac{I_{0}e^{-\beta V_{PDa}\left(0\right)}}{C_{PD}a}\left(1-e^{-\beta at}\right)}v_{PD}\left(t\right)+i_{n}\;\left(t\right)$$

Its solution is written as:

(32) $$v_{PD}\left(t\right)=\frac{1}{C_{PD}}\int_{o}^{t}dt\prime \frac{1+\frac{I_{0}e^{-\beta V_{PDa}\left(0\right)}}{C_{PD}a}\left(1-e^{-\beta at\prime }\right)}{1+\frac{I_{0}e^{-\beta V_{PDa}\left(0\right)}}{C_{PD}a}\left(1-e^{-\beta at}\right)}i_{n}\left(t\prime \right)+\frac{1}{1+\frac{I_{0}e^{-\beta V_{PDa}\left(0\right)}}{C_{PD}a}\left(1-e^{-\beta at}\right)}v_{PD}\left(0\right)$$

For Step 3, squaring Equation (32), averaging and using Equation (13), the tapered reset noise variance can be derived:

(33) $$<v_{PD}{\left(t\right)}^{2}>=\frac{mkT}{2C_{PD}}\left(1-\frac{1}{{\left(1+\frac{I_{a}\left(0\right)}{C_{PD}a}\left(1-e^{-\beta at}\right)\right)}^{2}}\right)+<v_{PD}{\left(0\right)}^{2}>\frac{1}{{\left(1+\frac{I_{a}\left(0\right)}{C_{PD}a}\left(1-e^{-\beta at}\right)\right)}^{2}}$$

If *a* is so small that $\beta at\ll 1,\;e^{-\beta at}\approx 1-\beta at,$ Equation (33) then becomes identical to that of the soft reset case, Equation (22). On the other hand, if *a* is enough large, $e^{-\beta at}$ decays so fast that the reset noise variance $<v_{PD}{\left(t\right)}^{2}>$ cannot reach the soft reset level. Therefore, the tapered reset shown in Figure 2c is not useful for noise reduction, although the average voltage $V_{PDa}\left(t\right)$, converges exponentially.

## 6. Feedback Reset with Reverse Taper Control (FRRT)

The feedback reset method was also proposed as a mean to reduce the reset noise [13,20,21,22,23,24,25,26]. In this method, during the reset period, the noisy PD voltage is detected and the resulting negative feedback forces the PD voltage to approach the reference level. A bidirectional current is needed at the RST for effective feedback, even though the subthreshold current is essentially unidirectional [13,24]. The concept of an “unidirectional current” means, in this context, that if the feedback (relaxation) times for upper and lower fluctuations are very different because of the current nonlinearity, the current looks unidirectional from the feedback point of view. One solution to overcome this contradiction is to constantly inject electrons into the PD; these injected electrons can then effectively play the role of a current flowing in the opposite direction. There are a couple of methods to perform this injection; one is to slowly ramp the RST gate toward the on-direction or positive direction [23,24], in contrast with what is done in the tapered reset method discussed in Section 5. Another method is to ramp the RST source voltage toward the on-direction or negative direction, as will be explained in detail in this section. It should be noted that electrons flow from the RD to the PD in both cases, regardless of the name of “drain”. Therefore, this method can be named feedback reset with reverse taper control (FRRT).

There is another important point to be considered when discussing reset noise reduction; excrescent noise should not be generated when the RST is turned off at the end of the reset period. If there are electrons at the RST channel just before it is turned off, these electrons are partitioned to the PD and the RD. This partitioning has a stochastic nature, and generates the partition noise [34]. Subthreshold operation tends to reduce this effect, because the electron number at the RST channel is smaller and unidirectional current is involved.

**Figure 3:**
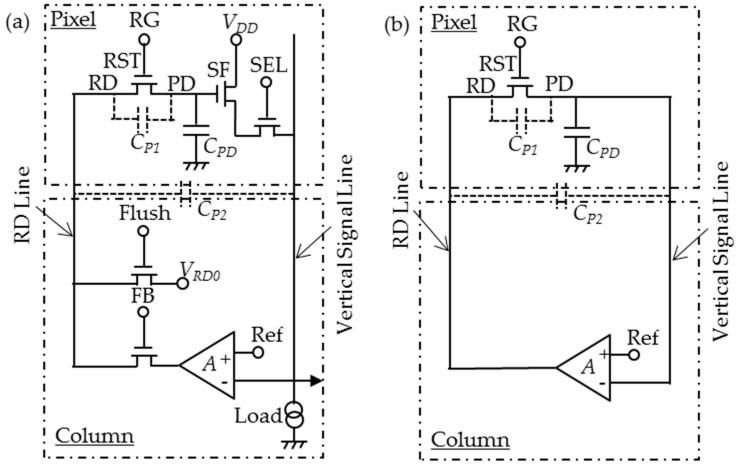
Feedback reset with reverse tapered control (FRRT); (**a**) Schematic; (**b**) Simplified model for noise analysis.

**Figure 4:**
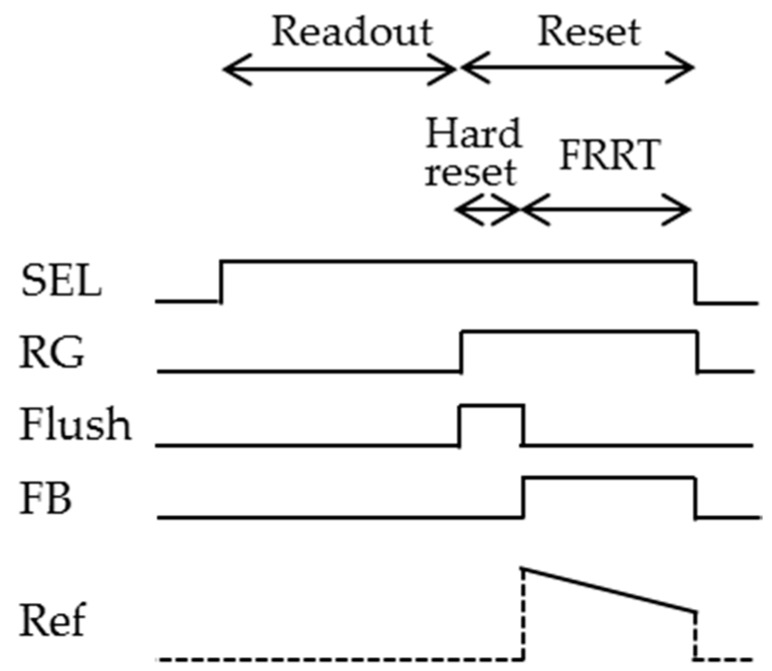
Timing diagram for one pixel in the case of feedback reset with reverse tapered control (FRRT).

Figure 3a shows a schematic diagram of the pixel and the related column-based feedback circuits for the FRRT to be analyzed in this section. The feedback is applied through the RD. The pixel structure is the same as the conventional 3-Tr scheme, as shown in Figure 1. The exception is that the RD wiring is prepared separately from the SF drain line. Other feedback circuits and ramp circuits are column-based. The vertical signal line, transferring the SF output voltage, is connected to the negative input of a column-based differential amplifier together with the load transistor and the following signal circuits. The positive input is connected with a ramp generator, $V_{Ref}=a_{0}-at$. The output of the amplifier is connected to the RD through the additional vertical RD line. Both the parasitic capacitance between PD and RD, *C_P1_*, and the parasitic capacitance between the vertical signal line and the RD line, *C_P2_*, are included in the analysis. It is assumed that *C_PD_* does not include *C_P1._* The timing chart for this structure is shown in Figure 4. After one row is selected by SEL, signal is read out in a fashion similar to the one of the conventional 3-Tr scheme at first. During the reset period, a hard reset is carried out in front to eliminate vestiges of previous signals, by setting Flush to ON. Subsequently, FRRT is executed turning FB ON and gradually decreasing $V_{Ref}$.

Figure 3b shows the simplified schematic diagram for noise modeling; the SF is merged with the high-gain differential amplifier, of gain *A*. The amplifier is also assumed to be faster than the reset motion. The parasitic capacitances and parasitic resistances (which delay the feedback) for both the vertical signal line and the RD line are neglected because fast feedback is assumed. The parasitic capacitances *C_P_*_1_ and *C_P_*_2_ are included because they have an important role in the feedback connecting the amplifier’s input and output. Only reset noise or RST channel noise is considered here; noises from the SF, the differential amplifier, SEL and wiring resistances are not included, because the reset noise is dominant.

The continuity equation becomes:

(34) $$(C_{PD}+C_{P1}+C_{P2})\frac{dV_{PD}\left(t\right)}{dt}-\left(C_{P1}+C_{P2}\right)\frac{dV_{RD}\left(t\right)}{dt}=-I_{a}\left(t\right)-i_{n}\left(t\right)=-I_{0}e^{-\beta V_{RD}\left(t\right)}-i_{n}\;\left(t\right)$$

(35) $$V_{RD}\left(t\right)=A\left(V_{Ref}\left(t\right)-V_{PD}\left(t\right)\right)=A\left(a_{0}-at-V_{PD}\left(t\right)\right)$$

Substituting Equation (35) into Equation (34), the equation for $V_{PD}\left(t\right)$ is obtained as:

(36) $$C_{T}\frac{dV_{PD}\left(t\right)}{dt}=-AC_{P}a-I_{0}e^{-\beta A\left(a_{0}-at-V_{PD}\left(t\right)\right)}-i_{n}\;\left(t\right)$$

where $C_{T}\equiv C_{PD}+\left(1+A\right)C_{P}\text{ and }C_{P}\equiv C_{P1}+C_{P2}$.

For Step 1, the continuity equation for average voltage $V_{PDa}\left(t\right)$, is:

(37) $$C_{T}\frac{dV_{PDa}\left(t\right)}{dt}=-AC_{P}a-I_{0}e^{-\beta A\left(a_{0}-at-V_{PDa}\left(t\right)\right)}$$

Equation (37) can be transformed to eliminate the constant term, as follows:

(38) $$C_{T}\frac{d(V_{PDa}\left(t\right)+\frac{AC_{P}a}{C_{T}}t)}{dt}=-I_{0}e^{-\beta A\left(a_{0}-a\frac{C_{PD}+C_{P}}{C_{T}}t-\left(V_{PDa}\left(t\right)+\frac{AC_{P}a}{C_{T}}t\right)\right)}$$

Its solution is:

(39) $$V_{PDa}\left(t\right)=-\frac{AC_{P}a}{C_{T}}t-\frac{1}{\beta A}log\left[e^{-\beta AV_{PDa}\left(0\right)}+\frac{I_{0}e^{-\beta Aa_{0}}}{a\left(C_{PD}+C_{P}\right)}\left(e^{t/\tau_{FRRT}}-1\right)\right]$$

where:

(40) $$\tau_{FRRT}\equiv \frac{1}{\beta Aa}\frac{C_{T}}{C_{PD}+C_{P}}$$

when t→∞, $V_{PDa}\left(t\right)$ approaches the asymptotic form exponentially:

(41) $$V_{PDa}\left(t\right)\to -at$$

This divergence is reasonable because of the substantial charge injection to the PD.

For Step 2, substituting Equation (39) into Equation (36) we obtain the equation for $v_{PD}\left(t\right)$ as:

(42) $$C_{T}\frac{dv_{PD}\left(t\right)}{dt}=\frac{I_{a}\left(0\right)e^{t/\tau_{FRRT}}\left(1-e^{\beta Av_{PD}\left(t\right)}\right)}{1+\frac{I_{a}\left(0\right)}{a\left(C_{PD}+C_{P}\right)}\left(e^{t/\tau_{FRRT}}-1\right)}-i_{n}\;\left(t\right)$$

Assuming that $\beta Av_{PD}\left(t\right)\;\ll 1,$ one can use the approximation:

(43) $$e^{\beta Av_{PD}\left(t\right)}\approx 1+\beta Av_{PD}\left(t\right)$$

The validity of this assumption will be discussed later. Equation (42) then becomes a linear equation as:

(44) $$C_{T}\frac{dv_{PD}\left(t\right)}{dt}=-\frac{\beta AI_{a}\left(0\right)e^{t/\tau_{FRRT}}v_{PD}\left(t\right)}{1+\frac{I_{a}\left(0\right)}{a\left(C_{PD}+C_{P}\right)}\left(e^{t/\tau_{FRRT}}-1\right)}-i_{n}\;\left(t\right)$$

The solution can be written as:

(45) $$v_{PD}\left(t\right)=-\frac{1}{C_{T}}\int_{o}^{t}dt^{\prime }\frac{1+\frac{I_{a}\left(0\right)}{a\left(C_{PD}+C_{P}\right)}\left(e^{t\prime /\tau_{FRRT}}-1\right)}{1+\frac{I_{a}\left(0\right)}{a\left(C_{PD}+C_{P}\right)}\left(e^{t/\tau_{FRRT}}-1\right)}i_{n}\left(t^{\prime }\right)+\frac{1}{1+\frac{I_{a}\left(0\right)}{a\left(C_{PD}+C_{P}\right)}\left(e^{t/\tau_{FRRT}}-1\right)}v_{PD}\left(0\right)$$

For Step 3, squaring Equation (45), averaging and using Equation (13), the noise variance can be obtained as:

(46) $$<v_{PD}{\left(t\right)}^{2}>=\frac{mkT}{2AC_{T}}\left(1-\frac{1}{{\left(1+\frac{I_{a}\left(0\right)}{a\left(C_{PD}+C_{P}\right)}\left(e^{t/\tau_{FRRT}}-1\right)\right)}^{2}}\right)\;+<v_{PD}{\left(0\right)}^{2}>\frac{1}{{\left(1+\frac{I_{a}\left(0\right)}{a\left(C_{PD}+C_{P}\right)}\left(e^{t/\tau_{FRRT}}-1\right)\right)}^{2}}$$

The first term is caused by shot noise, and the second term results from the initial condition. When $C_{P}=0$, the asymptotic form and the limit for *t*→∞ are obtained as:

(47) $$<v_{PD}{\left(t\right)}^{2}>\approx \frac{mkT}{2AC_{PD}}(1-e^{-2\beta Aat})+<v_{PD}{\left(0\right)}^{2}>e^{-2\beta Aat}$$

(48) $$<v_{PD}{\left(\infty \right)}^{2}>=\frac{mkT}{2AC_{PD}}$$

When $C_{P}\neq 0$, the asymptotic form and the limit for *t*→∞ become:

(49) $$<v_{PD}{\left(t\right)}^{2}>\approx \frac{mkT}{2A\left(C_{PD}+\left(1+A\right)C_{P}\right)}(1-e^{-2t/\tau_{FRRT}})+<v_{PD}{\left(0\right)}^{2}>e^{-2t/\tau_{FRRT}}$$

(50) $$<v_{PD}{\left(\infty \right)}^{2}>=\frac{mkT}{2A\left(C_{PD}+\left(1+A\right)C_{P}\right)}$$

Considering that the detection capacitance is $C_{PD}+C_{P1}$, the reset noise variance in electron numbers at the PD, $<n_{PD}{\left(\infty \right)}^{2}>$, is derived as:

(51) $$<n_{PD}{\left(\infty \right)}^{2}>=\frac{mkT{\left(C_{PD}+C_{P1}\right)}^{2}}{2q^{2}A\left(C_{PD}+\left(1+A\right)C_{P}\right)}$$

where q denotes the electronic elementary charge.

**Figure 5:**
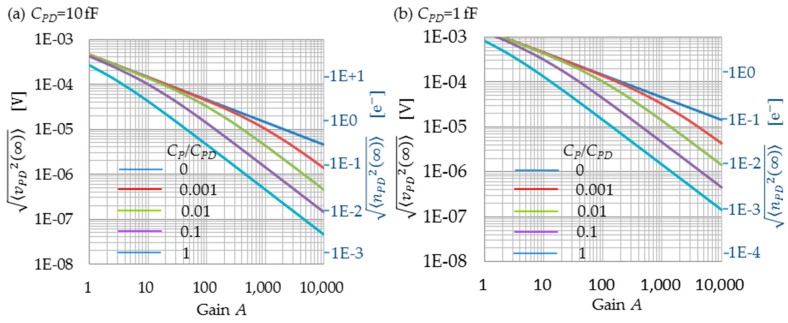
FRRT reset noise, with *C_P1_* = 1 fF. (**a**) *C_PD_* = 10 fF; (**b**) *C_PD_* = 1 fF. $<v_{PD}{\left(\infty \right)}^{2}>$ is the reset noise variance in voltage at the PD as expressed by (50), and $<n_{PD}{\left(\infty \right)}^{2}>$ is the reset noise variance in number of electrons at the PD as expressed by (51).

When $C_{P}=0$, the limit of the noise variance $<v_{PD}{\left(\infty \right)}^{2}>$ is 1/*A* times smaller than the soft reset noise variance, $\text{mkT}/2C_{PD}$, according to Equation (48). The reset noise is therefore much reduced. When $C_{P}\neq 0$, $<v_{PD}{\left(\infty \right)}^{2}>$ becomes even smaller than that when $C_{P}=0$, because *C_P_* couples the output of the amplifier to the PD directly, which contributes as a capacitive feedback [21], in addition to the feedback path through the RST. However, large values of *C_P_* have some drawbacks as well; if the vertical signal line capacitance and the RD line capacitance are large, the feedback speed is limited. If *C_P1_* is large, the conversion gain is decreased. Figure 5a,b show the reset noises for *C_PD_* = 10 fF and 1 fF, respectively. The horizontal axis denotes the amplifier gain *A*, the left vertical axis represents the reset noise voltage $\sqrt{<v_{PD}{\left(\infty \right)}^{2}>}$, and the right vertical axis represents the reset noise in number of electrons, $\sqrt{<n_{PD}{\left(\infty \right)}^{2}>}$. The parameter for the curves is $C_{P}/C_{PD}$. Here, *C_P1_* = 0 fF is assumed for simplicity. According to this simple model, the reset noise decreases as $A^{-1/2}$ when $C_{P}/C_{PD}$ = 0. When $C_{P}/C_{PD}$ ≠ 0, the reset noise decreases also as $A^{-1/2}$ for large values of *A*. It should be noted that even small values of $C_{P}/C_{PD}$ will drastically decrease the reset noise when *A* is larger. For example, when $C_{P}/C_{PD}=0.01$, the reset noise is decreased to 30% at *A* = 1000. If *C_PD_* + *C_P1_* is smaller, reset noise in number of electrons becomes smaller while the reset noise in voltage becomes larger. It is important to reduce the detection capacitance, *C_PD_* + *C_P1_*, as is also the case with the 4-Tr. scheme.

**Figure 6:**
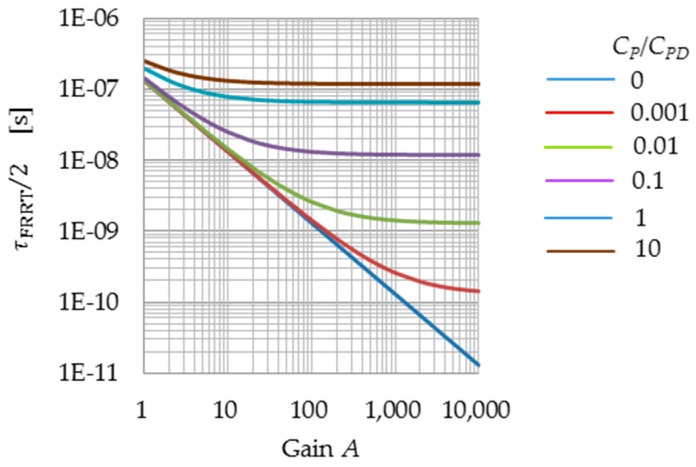
Time constant of the FRRT process (τ_FRRT_), for a=0.1 V/μs. $\frac{\tau_{FRRT}}{2}\equiv \frac{1}{2\beta Aa}\frac{C_{PD}+\left(1+A\right)C_{P}}{C_{PD}+C_{P}}.$

Even though FRRT uses a subthreshold current mode, $<v_{PD}{\left(t\right)}^{2}>$ still converges with fast exponential decay. Figure 6 shows the time constant, $\tau_{FRRT}/2$. In the figure, the horizontal axis represents the gain *A*, assuming that a=0.1 V/μs. When $C_{P}=0$, the time constant decreases linearly with 1/*A*. When $C_{P}\neq 0$, $\tau_{FRRT}/2$ decreases linearly with 1/*A* at *AC_P_* << *C_PD_*, while it becomes a constant with the value $\frac{C_{P}}{2\beta a\left(C_{PD}+C_{P}\right)}$ for *AC_P_* >> *C_PD_*. In the extremely unfavorable case of *C_P_*/*C_PD_* = 10, $\tau_{FRRT}/2$ is in practice sufficiently small (as small as 0.12 µs) for *A* > 20.

Using FRRT, the reset voltage is reduced by $aT_{FRRT}$, where *T_FRRT_* is FRRT period. This decreases the saturation of the PD. If a is increased $\tau_{FRRT}$ decreases as 1/a, and *T_FRRT_* can be decreased in the same manner. If *T_FRRT_* is adjusted properly, a will not affect the reset voltage reduction, because the reset voltage reduction dependence on a is given as $aT_{FRRT}\sim a^{0}$.

According to this simple model, if $A\to \infty ,\;<v_{PD}{\left(\infty \right)}^{2}>\to 0$. Limitations to this ideal case should be discussed below.

**Figure 7:**
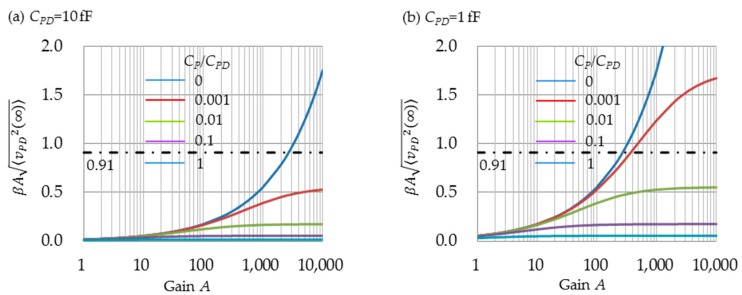
$\text{Depnedence of }\beta A\sqrt{\langle v_{PD}{}^{2}\left(\infty \right)\rangle }\text{ on gain }A\text{ and the ratio }C_{P}/C_{PD}$ If $\beta A\sqrt{\langle {v_{PD}}^{2}\left(\infty \right)\rangle }$ is smaller than 0.91, the approximation Equation (43) is permitted with an error of 30%.

Firstly, the approximation:

$$e^{\beta Av_{PD}\left(t\right)}\approx 1+\beta Av_{PD}\left(t\right)$$

is examined. If 30% of error is allowed, $\beta Av_{PD}\left(t\right)$ is limited by:

(52) $$1\leq \frac{e^{\beta Av_{PD}\left(t\right)}}{1+\beta Av_{PD}\left(t\right)}<1.3$$

which is always larger than 1. This means that $\beta Av_{PD}\left(t\right)$ should be smaller than 0.91. Figure 7a,b shows the exponent $\beta \text{A}\sqrt{<v_{PD}{\left(\infty \right)}^{2}>}$, substituting $\sqrt{<v_{PD}{\left(\infty \right)}^{2}>}$ to $v_{PD}\left(t\right)$ and using m=1 and 1/β=mkT/q=0.026 V. When $C_{P}=0$, the upper limit of *A* is obtained as 2,700 for *C_PD_* = 10 fF and as 270 for *C_PD_* = 1 fF, respectively. When $C_{P}\neq 0$, and because Equation (50) < Equation (48), the range within which approximation Equation (43) can be used becomes larger. For example, when $C_{P}/C_{PD}=0.01$, the upper limit becomes more than 10,000 for both *C_PD_* = 10 fF and *C_PD_* = 1 fF.

If the approximation Equation (43) becomes invalid, the quantitative discussion is difficult. However, the feedback effect becomes rather larger because the first term of right hand side at Equation (42) has larger negative value than that of Equation (44).

Secondly, the assumption that the feedback is faster than the reset motion should be discussed. Both the differential and SF amplifiers have finite output impedances, and both the vertical signal and RD lines have parasitic resistances and capacitances; this means that the feedback has a finite time constant. It increases as *A* increases. If the feedback becomes slow compared with the reset motion, the reset noise is increased in reverse. Therefore, the reset noise has a minimum at some value of *A*.

The dimensionless factor $I_{a}\left(0\right)/a\left(C_{PD}+C_{P}\right)$, at Equation (46) represents the ratio between the PD voltage change, $I_{a}\left(0\right)/\left(C_{PD}+C_{P}\right)$, and the taper slope, a. Assuming typical parameters: $I_{a}\left(0\right)=0.5\;\mu \text{A},\;C_{PD}=10\text{ fF},\;C_{P}/C_{PD}=0.01,\text{and }a=0.1\;V/\mu s$, we obtain $I_{a}\left(0\right)/a\left(C_{PD}+C_{P}\right)$ = 0.5, which is in the order of 1 and does not affect the convergence.

As discussed above, it can be said that FRRT reduces both reset noise and the convergence time constant. In fact, 2.5 $e^{-}$∙rms reset noise and 2.9 $e^{-}$ rms readout noise have been reported, using organic photoconductive film CMOS image sensor with 3 μm pixel, 5 μs reset period and *A* = 100 [13].

## 7. Electron Counting Possibility

Photon counting imaging is one of the grand targets for image sensor development. It requires two conditions; electron counting and high quantum efficiency [35]. In this section, the possibility of electron counting using FRRT is discussed, leveraging the good properties of the 3-Tr scheme discussed in Section 1. Before that, the statuses of other approaches, such as single-photon avalanche diode (SPAD) image sensors [36,37] and 4-Tr CMOS image sensors are reviewed.

The Geiger mode avalanche in SPADs creates a sharp spike signal from one original photon-generated electron-hole pair. The spike signal is so large that the in-pixel circuitry detects it as a digital signal and subsequent stages do not add any noises. The sharp spike also realizes time stamp, which is important for various applications such as time of flight, or fluorescence lifetime imaging microscopy (FLIM). Its weak points are the large dark count, the after pulse, and small fill factor because of the necessity of a guardring.

The 4-Tr CMOS image sensor saw some progress in 2015 [38,39,40,41,42], obtained mainly by reducing the detection capacitance or floating diffusion capacitance. It brings large conversion gain (as large as 426 $\mu \text{V}/e^{-}$) [41] and a readout noise as small as 0.27 $e^{-}\cdot$rms [42]. If the readout noise is less than 0.3 $e^{-}\cdot$rms, it can be said that elctron counting is possible with the 90% confidence level [35]. This method has a rather small dark current and a lager fill factor—even for small pixels—than SPADs. It also does not suffer from the after pulse. The 4-Tr scheme is not convenient for time stamping, because the pixel has to wait for a photon after holding the reset level for CDS and a longer period between reset sampling signal sampling makes the CDS 1/*f* noise reduction less effective. In contrast, the 3-Tr scheme is operated in a “signal-first, reset-later” mode, which is suitable for time stamping. 

With the FRRT simple model shown in the previous section, the reset noise, $\sqrt{<n_{PD}{\left(\infty \right)}^{2}>}$, becomes 0.10 $e^{-}\cdot$rmsfor $C_{PD}=10\text{ fF}$, $C_{P}/C_{PD}=0.01$, and *A* = 2,700, and becomes 0.29 $e^{-}\cdot$rms for $C_{PD}=1\text{ fF}$, $C_{P}/C_{PD}=0.01$, and *A* = 270. In those cases, the possibility of electron counting exists if the other noises are small enough.

The readout noise for the 3-Tr scheme is constituted by the reset noise, SF thermal noise, SF 1/*f* noise, column circuit noise, and ADC quantization noise. The most effective method to reduce these noises is to reduce the detection capacitance and to increase the conversion gain as done in the 4-Tr scheme; the noises will then be reduced in terms of the number of electrons at the PD. There is, however, a sharp tradeoff between the reduction of the detection capacitance and sensitivity, because using a smaller PD area to decrease the detection capacitance originates also a small sensitivity. Therefore, it is much difficult to achieve an electron counting capability with a 3-Tr scheme than with a 4-Tr scheme. One possible circuit-based approach is to combine a capacitive transimpedance amplifier [27,28] with the FRRT.

Another possibility is to reduce the SF thermal noise, SF 1/*f* noise, column circuit noise, and ADC quantization noise themselves. The SF thermal noise, column circuit noise, and ADC quantization noise could be reduced by circuit technologies. Although various methods have been reported to reduce the SF 1/*f* noise, additional improvements are needed to perform the electron counting with a 3-Tr scheme.

## 8. Conclusions

To discuss the reset noise generated by a slow subthreshold current, intuitive and simple analytical forms are derived in spite of the subthreshold current nonlinearity, which characterize the time evolution of the reset noise during the reset operation.

For soft reset, the reset noise limit when t→∞, $\sqrt{<v_{PD}{\left(\infty \right)}^{2}>}$, is given by $\sqrt{mkT/2C_{PD}}$, which agrees with previous published works. The asymptotic time dependence of the noise, $\sqrt{<v_{PD}{\left(t\right)}^{2}>}$, decreases with $t^{-1}$, even though the asymptotic time dependence of the average PD voltage, $V_{PDa}\left(t\right)$, is as slow as logt. The flush reset method is effective because the hard reset part eliminates the image lag, and soft reset part reduces the noise to the soft reset noise level.

The tapered reset method achieves exponential convergence, but the reset noise reduction is insufficient.

Finally, the FRRT shows both a fast convergence and a good reset noise reduction. When *A* is large, even small values of $C_{P}/C_{PD}$ can drastically decrease the reset noise. If the feedback is sufficiently fast, the reset noise limit when t→∞, $\sqrt{<n_{PD}{\left(\infty \right)}^{2}>}$, becomes $\frac{mkT{\left(C_{PD}+C_{P1}\right)}^{2}}{2q^{2}A\left(C_{PD}+\left(1+A\right)C_{P}\right)}$. Assuming that $C_{PD}=10\text{ fF}$, $C_{P}/C_{PD}=0.01$ and *A* = 2700, $\sqrt{<n_{PD}{\left(\infty \right)}^{2}>}$ becomes 0.10 $e^{-}$∙rms according to this simple model. Achieving an electron counting capability with this architecture requires a challenging 1/*f* noise reduction, even if the reset noise can be decreased.

## Abbreviations

The following abbreviations are used in this manuscript:

Tr	Transistor
CMOS	Complementary metal oxide semiconductor
PPD	Pinned photodiode
CDS	Correlated double sampling
AEC	Auto exposure control
PD	Photodiode
UV	Ultraviolet
IR	Infrared
Ge	Germanium
InGaAs	Indium-gallium-arsenide
RST	Reset transistor
SF	Source follower
SEL	Row select transistor
*k*	Boltzmann constant
*T*	Absolute temperature
*C*	Capacitance
$e^{-}$	Electron
CCD	Charge coupled devices
RD	Reset transistor drain
FRRT	Feedback reset with reverse taper control
FB	Feedback
Ref	Reference
SPAD	Single photon avalanche diode
FLIM	Fluorescence lifetime imaging microscopy
rms	Root mean square
